# Emerging Promise of Therapeutic Approaches Targeting Mitochondria in Neurodegenerative Disorders

**DOI:** 10.2174/1570159X21666230316150559

**Published:** 2023-04-12

**Authors:** Md. Mominur Rahman, Mst. Afroza Alam Tumpa, Md. Saidur Rahaman, Fahadul Islam, Popy Rani Sutradhar, Muniruddin Ahmed, Badrah S. Alghamdi, Abdul Hafeez, Athanasios Alexiou, Asma Perveen, Ghulam Md. Ashraf

**Affiliations:** 1 Department of Pharmacy, Faculty of Allied Health Sciences, Daffodil International University, Dhaka, Bangladesh;; 2 Department of Physiology, Faculty of Medicine, King Abdulaziz University, Jeddah, Saudi Arabia;; 3 Pre-Clinical Research Unit, King Fahd Medical Research Center, King Abdulaziz University, Jeddah, Saudi Arabia;; 4 The Neuroscience Research Unit, Faculty of Medicine, King Abdulaziz University, Jeddah, Saudi Arabia;; 5 Glocal School of Pharmacy, Glocal University, Mirzapur Pole, Saharanpur, Uttar Pradesh, India;; 6 Department of Science and Engineering, Novel Global Community Educational Foundation, Hebersham, Australia;; 7 AFNP Med Austria, Wien, Austria;; 8 Glocal School of Life Sciences, Glocal University, Mirzapur Pole, Saharanpur, Uttar Pradesh, India;; 9 Department of Medical Laboratory Sciences, College of Health Sciences, and Sharjah Institute for Medical Research, University of Sharjah, Sharjah, 27272, United Arab Emirates

**Keywords:** Mitochondria, neurodegenerative disorders, Parkinson’s disease, Alzheimer’s disease, antioxidants, gene therapy

## Abstract

Mitochondria are critical for homeostasis and metabolism in all cellular eukaryotes. Brain mitochondria are the primary source of fuel that supports many brain functions, including intracellular energy supply, cellular calcium regulation, regulation of limited cellular oxidative capacity, and control of cell death. Much evidence suggests that mitochondria play a central role in neurodegenerative disorders (NDDs) such as Parkinson’s disease, Alzheimer’s disease, Huntington’s disease, and amyotrophic lateral sclerosis. Ongoing studies of NDDs have revealed that mitochondrial pathology is mainly found in inherited or irregular NDDs and is thought to be associated with the pathophysiological cycle of these disorders. Typical mitochondrial disturbances in NDDs include increased free radical production, decreased ATP synthesis, alterations in mitochondrial permeability, and mitochondrial DNA damage. The main objective of this review is to highlight the basic mitochondrial problems that occur in NDDs and discuss the use mitochondrial drugs, especially mitochondrial antioxidants, mitochondrial permeability transition blockade, and mitochondrial gene therapy, for the treatment and control of NDDs.

## 1 INTRODUCTION

The mitochondrion, known for its role in the development of energy production, is an important intersection of biochemical, psychological, and social factors that influence the nature of the stress response and outcomes [1]. Mitochondria are the only organelles that have their own non-nuclear DNA. Mitochondrial DNA (mtDNA) consists of 16,500 circular base pairs and 37 genes. Genetic and biological instability is present in the maternal inheritance of mitochondria [2, 3]. Mitochondria can replicate, transcribe, and decode their DNA independently of nuclear DNA and may originate from isolated species that have become part of the cell. Nevertheless, it is dependent on cellular activity and mitochondrial functions [4]. Mitochondrial structure and function are artfully receptive to the environment and function both as stress response targets and mediators. As an energy producing organelle, mitochondria are particularly densely populated in the brain. There is, however, a significant influence of mitochondrial dysfunction on psychological functioning, and a growing body of research suggests that correlations exist between mitochondrial dysfunction, stress, and psychopathy [1, 5]. Mitochondrial disorders is a general term for many different clinical conditions that are associated with the general characteristics of mitochondrial dysfunction and abnormal energy metabolism. Mitochondrial disorders can occur at any age in either the nuclear genome (nDNA) or the mitochondrial genome and are due to gene mutations [6, 7]. Mitochondrial disturbance is the most common molecular theme in neurodegenerative disorders (NDDs). It is suggested that the mitochondrial perturbations detected are consistent with mitochondrial dysfunction [8]. In NDDs, the role that mitochondrial dysfunction plays is often uncertain and may vary from disorder to disorder. In certain disorders, it leads to neuronal dysfunction and, eventually degeneration. In others, it may play an intermediate but unique correlative role [9-11].

The identification of NDDs as mitochondrial disorders has gained increasing interest in recent years. Several NDDs, including Alzheimer’s disease (AD), Parkinson’s disease (PD), Huntington’s disease (HD), amyotrophic lateral sclerosis (ALS), and progressive supranuclear palsy, have been found to involve free radicals, mtDNA mutations, reduced mitochondrial respiration, and mitochondrial calcium dysregulation to varying degrees [10-14]. Considering the widespread mitochondrial pathology, mitochondrial dysfunction is thought to be involved in the pathophysiological mechanism of neurological disorders. It has also been suggested that clinical approaches to treat NDDs that target mitochondrial dysfunction should be developed [15-17].

Recent developments in genetic, biological, biochemical, and animal model studies of hereditary NDDs have shown that mutant proteins-such as amyloid-beta (Aß) in AD, mutant huntingtin in HD, and mutant parkin and a-synuclein in PD-are correlated with mitochondria, leading to increased free radical production, low cellular adenosine triphosphate (ATP) production, and ultimately cell death [18].

Mitochondrial dysfunction is now recognized as a typical cellular alteration in the progression of many hereditary NDDs. The importance of developing therapeutics to treat mitochondria in age-related NDDs is underscored by the role of mitochondrial dysfunction in both hereditary and late-onset NDDs. In this article, we discuss the physiological and functional changes of mitochondria in the elderly and patients with age-related NDDs. This article also discusses the potential of mitochondrial drugs for the treatment of aging and NDDs. Research that has uncovered mitochondrial characteristics associated with cellular dysfunction NDDs is reviewed in this article. The defects of the mitochondria and potential mitochondrial treatments for NDDs are also covered in this paper. Here, we describe some current applications of mitochondrial medicine in the treatment of NDDs and provide an overview of mitochondrial pathologies in NDDs. As scientific evidence that mitochondrial medicine shows promise in the treatment of NDDs, in this article, we discuss recent developments in a number of mitochondrial therapies, including antioxidants, mitochondrial permeability transition blocking, and mitochondrial gene therapy.

## 2 MITOCHONDRIAL STRUCTURE AND FUNCTION

Since it has become established that mitochondria are involved in a variety of cellular processes, such as metabolite formation and signal transduction, in addition to their conventional role in energy metabolism, they are no longer considered merely the powerhouse of the cell [19]. Mitochondria have greatly enhanced the energy production of the cell and generate ATP through the respiratory chain [20]. mtDNA is a double-stranded molecule that lacks introns and is polycistronic, just like bacterial DNA. Most mitochondrial genes have been lost or transferred to nuclear DNA during evolution, leaving mtDNA with only 37 genes: 11 messenger ribonucleic acids (mRNAs), which are translated into 13 proteins, 2 ribosomal (RNAs) (rRNAs, 12S, and 16S), and 22 transfer RNAs (tRNAs) [21].

**Fig. (1):**
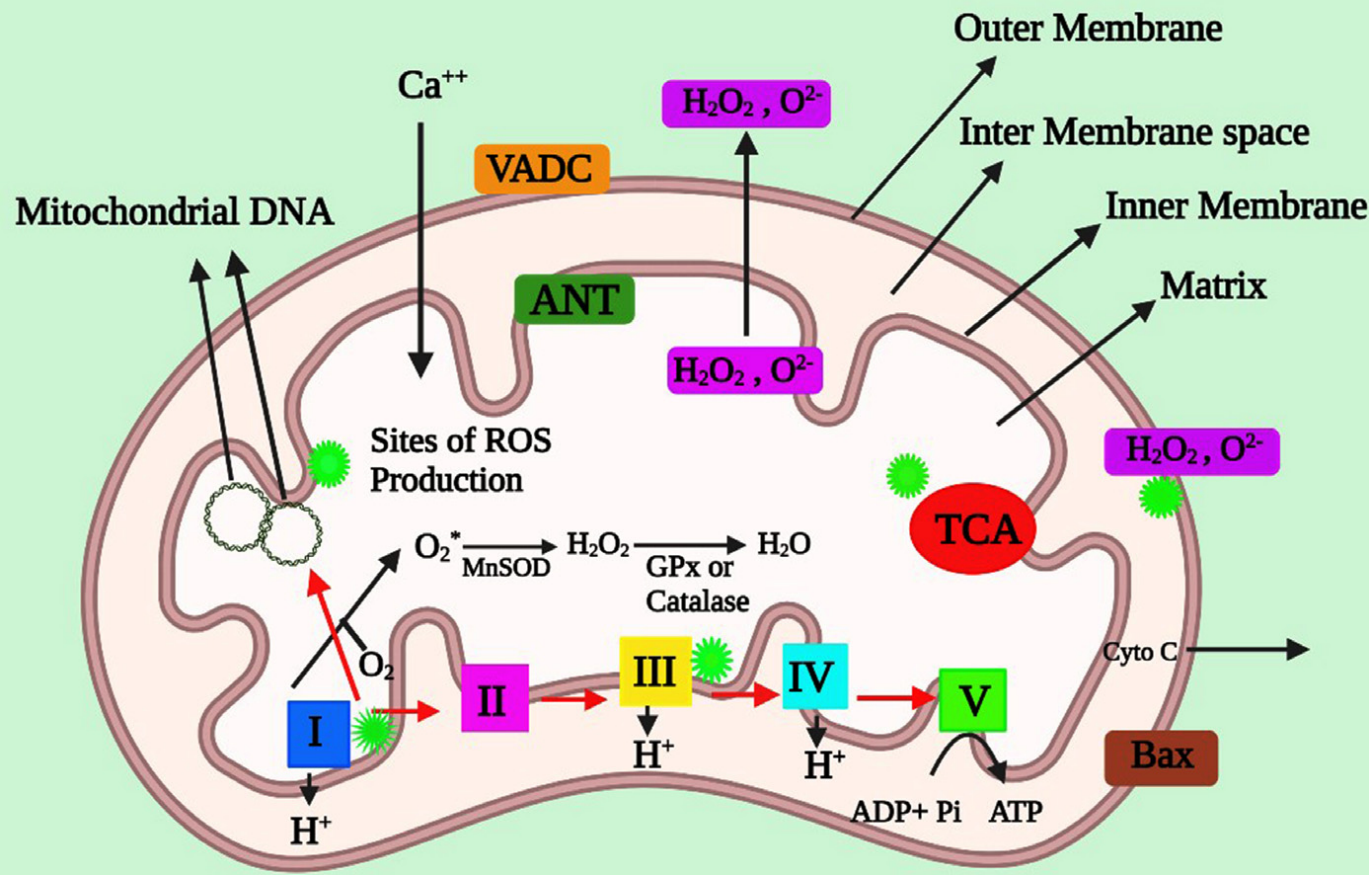
The structure of mitochondria and the locations where free radicals are produced. Two lipid membranes separate a mitochondrion: the inner mitochondrial membrane and the outer mitochondrial membrane. The mitochondrial respiratory chain is housed in the inner mitochondrial membrane and serves as a highly effective barrier to ion flow. A mitochondrial genome is located in 2-10 copies in each mitochondrion. Complexes I and III of the electron transport chain (ETC) lose electrons to oxygen, producing superoxide radicals. Manganese superoxide dismutase produces H_2_O_2_ by dismutating superoxide radicals. ETC also includes catalase or other enzymes that convert H_2_O_2_ to H_2_O and O_2_. Glutathione peroxidase accepts electrons from NADH and FADH_2_ and uses them to generate ATP from adenosine diphosphate and inorganic phosphate. In the matrix, tricarboxylic acid also produces free radicals. These radicals are transported *via* voltage-dependent anion channels into the cytoplasm, where they can oxidize DNA and proteins. (*A higher resolution/colour version of this figure is available in the electronic copy of the article*).

The mitochondrial respiratory chain is housed in the inner mitochondrial membrane, which serves as an effective ion barrier. The mitochondrial matrix, which includes tricarboxylic acid and beta oxidation, is covered by the inner mitochondrial membrane. The outer mitochondrial membrane is porous, allowing low molecular weight molecules to flow between the cytosol and the intermembrane gap (Fig. **1**) [22]. The outer mitochondrial membrane (OMM) and inner mitochondrial membrane (IMM) envelop the mitochondria and divide the organelle into two regions, the intermembrane space (IMS) and the matrix [23]. Because of the endosymbiotic origin of the organelle, the two membranes have significant differences in lipid content, transmembrane protein properties and functions, permeability, and shape. The IMM has a larger protein/lipid ratio and forms cristae, which are densely packed invaginations in the matrix [24].

As mentioned earlier, the permeability of the OMM and the IMM differs significantly. The OMM allows ions and small molecules to flow through voltage-dependent anion channels (VDACs), whereas the IMM allows only water, oxygen (O_2_), and carbon dioxide (CO_2_) to pass freely. This selectivity allows the development of an electrochemical gradient across the membrane that is required for ATP generation and precise control of other ion concentrations, such as calcium, which is important for cell signaling [24, 25]. Internally, mitochondria are structured as cristae, which are invaginations of the IMM that may be dynamically reconfigured in response to different stimuli, such as changes in energy demand or apoptotic signals, by becoming more or less compact [26].

Because of their function in energy production, mitochondria are often referred to as the “powerhouse of the cell”. However, in the last 30 years, mitochondria have also been identified as signaling organelles involved in several physiological processes, including calcium homeostasis, apoptosis, and the production of heme and iron-sulfur clusters. Mitochondria play an important role in the metabolism of all mammalian cells, including neurons in the brain. Abnormalities in mitochondrial structure and function may cause age-related NDDs [27].

### 2.1 Mitochondrial Synthesis, Lifespan, and Decay

Mitochondria are eukaryotic organelles derived from free-living α-proteobacteria [28]. Mitochondria are found in almost every eukaryotic cell, including neurons. Neuronal mitochondria have a half-life of around one month [29]. All cells, including neurons, are involved in the breakdown of existing mitochondria and the synthesis of new mitochondria. The function of mitochondria in neurons is maintained by constant mitochondrial recycling [30]. The specific processes by which mitochondrial synthesis occurs are still unknown. There is evidence that mtDNA divides, facilitates mitochondrial replication, and completes mitochondrial synthesis. Mitochondria also grow from pre-existing mitochondria through mitochondrial fission, which occurs in all eukaryotic cells, including neurons. The functional advantages of the endosymbiont, particularly the ability to compartmentalize energy conversion *via* oxidative phosphorylation (OXPHOS), lay the foundation for the future success of eukaryotes. During evolution, genetic information from the endosymbiont to the nucleus was either lost or transmitted in an imperfect process, leaving mitochondria with only a vestigial genome [31].

Mitochondria are the primary source of reactive oxygen and nitrogen species (RONS) linked to aging, neurodegeneration, and apoptosis in cells and organisms [32]. Mitochondria are also involved in the control of apoptosis, or programmed cell death, which is essential for embryonic development and a variety of physiological activities. Apoptosis is the regulated and programmed death of cells that occurs as a result of various damages or stresses such as DNA damage, oxidative stress, immunological responses, and the absence of certain growth factors, hormones, and cytokines or as a natural part of development and aging [33]. Mitochondrial dysfunction has been identified as a key component in the etiology of many age-related neurodegenerative disorders [34]. Mitochondrial damage may impair neurotransmission and thus be responsible for clinical symptoms in neurodegenerative disorders.

### 2.2 Mitochondria in Neural Cells: Bioenergetics and Dynamics

Mitochondria are multifunctional organelles that evolved from prokaryotic bacteria by endosymbiotic evolution [35, 36]. The organelles are the primary source of reactive oxygen species and antioxidant defense of the cell, in addition to their essential bioenergetic role of ATP regeneration [37]. Mitochondrial function and structure are inextricably linked. Even in unicellular organisms such as yeast, mitochondria are complex and dynamically active. A growing number of studies show that variations in mitochondrial size and shape, collectively known as mitochondrial dynamics, are controlled by organelle fission and fusion events and play a key role in modulating mitochondrial function. Therefore, a mismatch between mitochondrial fusion and fission affects a wide variety of cellular biological activities [38].

Mitochondria are dynamic organelles composed of a complex network of tube-like structures [39]. Mitochondrial dynamics include mobility, fusion and fission, cristae modification, biogenesis, and mitophagy. The overall mitochondrial morphology changes in response to changing energy demands and cellular stress through these quality control mechanisms, which are tightly controlled and corrected [40].

NDDs, including AD, PD, and HD, and ALS are heterogeneous, insidious, and irreversible disorders that have distinct molecular and clinical features of deteriorating bioenergetics and abnormalities [41]. Deficits in bioenergetics are associated with mitochondrial dysfunction, such as deficiency of mitochondrial complexes I, III, and COX in AD, deficiency of mitochondrial complexes I and IV in PD, deficiency of mitochondrial complexes II, III, and IV in HD, and deficiency of mitochondrial complexes I, II, III, and IV in ALS. Therefore, new therapeutic strategies targeting neuronal bioenergetics are needed [42].

#### 2.2.1 The Mitochondrion: a Bioenergetic Engine

Progress in the field of mitochondrial bioenergetics has been strongly influenced by the work of leading scientists in the late 1940s and 1950s, such as Boyer *et al*., who proposed the “chemical-coupling hypothesis”. The theory of energy conversion in oxidative phosphorylation was challenged and replaced by Mitchell in the 1960s and later extended to energy conversion in bacteria and chloroplasts in addition to molecular proton pumps. After the identification of the most reactive mechanism, emphasis was placed on resolving the formation of complex molecular clusters, such as cytochrome c oxide, complex III, complex II, ATP synthesis, photosystem I, photosystemic water separation centers, and energy collection antennas of various photosynthetic systems. Modern trends have raised concerns about the reactivity of radicals and other active species involved in the bioenergetic activity. A promising trend is a possibility of focusing on the redox status of the cell [43].

#### 2.2.2 The Mitochondrial Electron Transport Chain

The mitochondrial ETC comprises complexes I-IV and the electron transporters ubiquinone and cytochrome c. The ETC has two electron transport pathways: complexes I/III/IV with nicotinamide NADH as a layer and succinic acid as complexes II/III/IV layer. Electron flow is combined to form a proton gradient across the inner membrane, and the energy accumulated in the proton gradients is used to produce ATP by complex V (ATP synthase) [44].

#### 2.2.3 The Mitochondrial Biosynthesis of ATP

The process of ATP synthesis appears to proceed as follows. When hydrogen atoms are transferred from FMNH2 or FADH2 to oxygen, protons (H^+^ ions) are pumped from inside the mitochondria to outside the cristae. In this way, respiration generates an electric potential (and a slight pH gradient of 200 to 300 millivolts across the membrane) in the mitochondria. The chemical energy of the layer is converted into electrical energy. A complex enzyme (ATP synthetase) is attached to the cristae that bind ATP, adenosine diphosphate (ADP) and inorganic phosphate (PI). It consists of nine polypeptide chain subunits of five different types in a cluster and a unit of at least three other membrane proteins that form ADP and PI's attachment points. This complex forms a specific proton pore in the membrane. When ADP and Pi bind to ATP synthase, protons (H^+^) formed outside the mitochondria return to the mitochondria through the enzyme complex. The energy released is used to convert ADP and PI into ATP. This process converts electrical energy into chemical energy, and the supply of ADP limits the rate of this process. The exact process by which the ATP synthase complex converts the energy stored in the electrical H^+^ gradient to chemical bonding energy in ATP is not well understood. In addition to ATP synthesis by the H^+^ gradient, it may also drive other endergonic (energy-consuming) processes, such as the movement of bacterial cells and the transport of carbon levels or ions [45].

#### 2.2.4 Modulation of Mitochondrial Fusion and Fission Dynamics

Mitochondria are dynamic organs that constantly merge and subsequently disintegrate. Defective or redundant mitochondria to maintain mitochondrial homeostasis are usually eliminated by a form of autophagy called mitophagy. Neurodegenerative disorders may be caused by imbalances in mitochondrial fission and fusion. Mitochondrial dynamics are tightly controlled by processes such as fusion and fission. The effectiveness of mitochondrial dynamics can lead to abnormal accumulation of mitochondria and contribute to cellular damage. Neurons are among the types of cells that consume the most energy, have a highly complex morphology, and are particularly dependent on mitochondrial function and motility [46].

## 3 MITOCHONDRIAL PATHOLOGY IN NEURODEGENERATIVE DISEASES

Neurons have synaptic terminals that propagate from the neuronal soma through the extension of long projections that fluctuate to form neuronal connections and provide objective neuronal terminal receptors and canals that facilitate neuronal functions. Mitochondria play a role in energy supply [47] and regulation of calcium kinetics and metabolism to meet the high energy demands of neurons [12]. With the prolonged duration of neural projection, the journey of mitochondria to their endpoint is prolonged and damaged mitochondria are at higher risk of injury [48]. The high energy demand of neurons increases the risk of oxidative mitochondrial stress. Because neural mitochondria are both active and severely damaged, it is not surprising that mitochondria are the link between various neuronal disorders and pathologies. Mitochondria have been implicated in the physiopathological mechanism of various NDDs, including AD and PD. Mitochondrial dysfunction prevalent in various NDDs includes, to varying degrees, severe oxidative stress, mutations in DNA, low ATP production, calcium homeostasis dysfunction, mitochondrial permeability transition, lack of mitochondrial movement, as well as an imbalance between fusion and fission of mitochondria [12, 49].

### 3.1 Mitochondrial Respiratory Complexes

Mitochondrial respiratory complexes comprise nicotinamide adenine dinucleotide dehydrogenase-ubiquinone oxidoreductase, succinate dehydrogenase, ubiquinol-cytochrome c oxidoreductase, and cytochrome c oxidase, which are known as complexes I to IV, respectively [50]. During electron transport, a proton gradient is passed through the mitochondrial respiratory complexes; thus, ATP is produced during the oxidative process of phosphorylation. In addition, mitochondrial complexes I and III have been identified as crucial parts of mitochondrial free radical production. The weakened activities of complexes II and IV contribute to the pathological generation of reactive oxygen species (ROS) [51]. In respiratory disorders, the mitochondrial complexes expand the electron leakage from ETC, allowing electrons to accumulate, which exacerbates the current cellular/mitochondrial disruptions of free radicals and ROS. When something goes wrong, the direct effects of mitochondrial respiratory damage on the chain lead to decreased ATP production and increased free radical accumulation in mitochondria, both of which are common pathologies in NDDs. Abnormalities of mitochondrial respiratory complexes are inhibited in certain neurodegenerative diseases. Deactivation of the mitochondrial complex is common in the substantial nigra of PD patients, an observation consistent with animal models treated with 1-methyl-4-phenyl-1, 2, 3, 6-tetrahydropyridine (MPTP). Moreover, the reduced movement of mitochondrial complex I has been shown in lymphocytes and skeletal muscle and found in mitochondria and platelets from patients with HD and PD [52]. The functions of complexes II/III are decreased, and reduced platelet mitochondria have been documented in patients with sporadic PD [53, 54]. Such findings suggest dysfunction of the mitochondrial respiratory complexes. Using cytoplasmic hybrid (cybrid) technologies, many groups have recorded reduced mitochondrial complex I movement in cybrids with postsynaptic potential (PSP) [55].

Decreased complex I has also been observed in HD patients [56-58]. Symptomatic HD patients with complex II show decreased needs for surgery [55]. Symptomatic HD patients also show reduced activity of complex II iron-sulfur subunit in caudate and putamen tissues [59, 60]. The activity of complex III was also found in mitochondria derived from pretended brain regions and platelets from AD and Down syndrome patients [61, 62]. Besides, decreased complex IV was found in hippocampal mitochondria isolated from the temporal pole of patients with AD [63, 64]. AD subhuman models, AD cybrid cells, and cells treated with A beta also showed impaired complex IV activity [65-68]. It is unexpected that ATP generation was decreased due to a deficiency of mitochondrial respiratory complexes. Similar abnormalities are also observed in the temporal cortex as well as the hippocampus of patients with AD, and in the spinal cord of patients with ALS. Besides, reduced ATP concentration is observed in AD, PD, HD, and ALS animal or cell models, further supporting the role of ATP depletion in NDDs [69-71].

### 3.2 Reduced Ability of Mitochondria to Eliminate Radicals

Mitochondrial antioxidant protection setup, which is effective for the clearance of endogenous radicals produced by mitochondria, consists of small reducing molecules along with reduced glutathione (GSH), ubiquinone, ascorbic acid and tocopherols, as well as oxidation-catalyzing enzymes such as copper/zinc superoxide dismutase (SOD), manganese SOD, catalase, and glutathione peroxidase. There is increasing evidence that the potential of mitochondria in radical removal is reduced in NDDs. This is indicated by the observation that SOD1 accumulation occurs in the mitochondria of patients with familial and sporadic ALS. Similarly, a mitochondrial alteration observed in a case of familial ALS involves a mutation in SOD1 that results in reduced SOD activity in eliminating Cu/Zn [72, 73]. Furthermore, mice with transgenic SOD1 mutant close resembles ALS, further supporting the role of reduced SOD1 function in ALS pathogenesis [74]. Mitochondria are regulated by fission and fusion events to control various functions, including cell proliferation, neurotransmission, and apoptosis, and are associated with significant brain damage in AD [68, 74-77].

### 3.3 Mitochondria DNA (mtDNA) Lesions

The obvious function of mtDNA is to preserve the identity of mitochondria relative to other organelles in the cell. The mitochondrion consists of 2-10 copies of circular DNA that encodes 13 proteins associated with mitochondria, 2 ribosomal RNAs, and 22 tRNAs. mtDNA is inherited and is at high risk for lesion growth caused by several factors, the most common of which is oxidative stress because mtDNA is located in close proximity to sites where radicals are generated [78, 79]. There are various forms of mtDNA deficiency in NDDs, apart from point mutations, nucleic acid modifications, large-scale deletions, and reduced copies of mtDNA [80-86]. mtDNA plays a role that is still debated. The question is whether mDNA plays a role in intermittent NDDs. Studies in PD cybrids show that mtDNA deletion in neurons results in complex mitochondrial defects [87, 88]. The results of studies in cybrids suggest that PD and AD have unique mtDNA mutations in some mitochondrial respiratory complexes that correlate with defects [66].

However, several studies indicating that mtDNA damage is the same in patients with NDDs compared to control subjects of the same age have challenged this theory. They suggest that the distribution and toughness of mtDNA lesions in many NDDs are probably not specific to the disease but may not be independent of the pressure to which the damaged neurons are exposed [89, 90].

### 3.4 Mitochondrial Calcium Dyshomeostasis and Mitochondrial Permeability Transition Pore

Mitochondria are important organelles for modulating intracellular calcium homeostasis [91, 92]. Disruption of mitochondrial calcium balance in NDDs is a hallmark of mitochondrial pathology. In patients with AD, PD, HD, and ALS, as well as in animal/cell models of these diseases, mitochondrial calcium load is increased in affected areas, and the buffering capacity of the mitochondrial calcium is decreased [93-95]. To date, there is insufficient evidence that calcium disturbance is an underlying cause of NDDs, although a growing number of studies suggest that disrupted calcium metabolism plays a role in the disease pathogenesis along with free radicals, mitochondrial respiratory complex deficiency, apoptosis, *etc.* For example, αβ leads to an increased concentration of cytoplasmic calcium, resulting in mitochondrial calcium overload [96]. The comprehensive structure of MPTP is still debated and has not yet been elucidated. However, it is widely agreed that the basic composition of MPTP consists of the mitochondrial voltage-dependent anion channel (VDAC) and IMM [97]. Recently, studies have shown that phosphate carrier (PiC) may also be involved in the composition of MPTP [98]. The physiological feature of MPTP is unclear, but it is considered to be a gateway for other pathological conditions [97]. It leads to oxidative stress, mitochondrial calcium and phosphate overload, and cyclophilin D (CypD) translocation to the IMM [98, 99]. Indeed, the first step in the assembly of MPTP is the translocation of CypD to bind to an adenine nucleotide (ANT). After binding, ANT forms a tube by changing its shape with CypD. The channel formed by ANT and the channel composed of VDAC form a nonselective pore in both mitochondrial membranes [100, 101]. Massive MPTP disruptions in neurons have been demonstrated in parts of numerous neurodegenerative diseases, consistent with the hypothesis that abundant endogenous ROS development and calcium overload are normal pathological changes in NDDs. Thus, CypD levels were significantly elevated in AD-affected areas but not in AD patients. In addition to aged mice, the brains of transgenic AD mice, including the hippocampus and cortex, also show upregulation of CypD [102]. In the G93A-mSOD1 ALS mouse model, recent experiments have shown that immunostaining of CypD in motor neurons is associated with mitochondrial remodeling of IMM and matrix vesiculations, even in presymptomatic animals in the spinal cord. In contrast, genetic loss of CypD delayed the development of the disease and significantly prolonged survival in ALS mice [103]. Increased CypD expression levels and decreased MPTP thresholds are observed in a study in which CypD expression is induced in the rat striatum and other brain parts. Based on the susceptibility to MPTP comparisons and a mitochondrial abnormality, increased levels of CypD are suspected in the part of the brain affected by the disease. A possible risk for the etiology of HD could be this partial difference in CypD expression [104]. In addition, MPTP formation and reduced mitochondrial processing of calcium have been reported in various HD animal models, in the striatal cell line STHdhQ111/Q111, and in lymphocytes from HD patients [105].

## 4 MITOCHONDRIAL EFFECT ON THE HUMAN HORMONE

Studies have shown that mitochondria play an important role in many aspects of cell physiology, including calcium homeostasis, metabolism, ATP production, and apoptosis [106]. Mitochondria are also a very important organelle in the biosynthesis of steroid hormones, including the major female hormone, estrogen [107]. Estrogens are involved in many physiological functions, such as increasing cerebral blood flow, preventing atrophy of cholinergic neurons, and modulating trophic factors in the brain [108]. Estradiol is the most ubiquitous form of estrogen, which is 10 times more potent than estrone and about 80 times more potent than estriol in estrogenic action. Hormonal insufficiency occurs in menopausal women. The level of estrogen in the bloodstream is characterized by a sudden decrease during the reproductive phase [107, 109].

### 4.1 Mitochondrial Impairments in Brain Aging: Insight into the Role of Estrogen

**Fig. (2):**
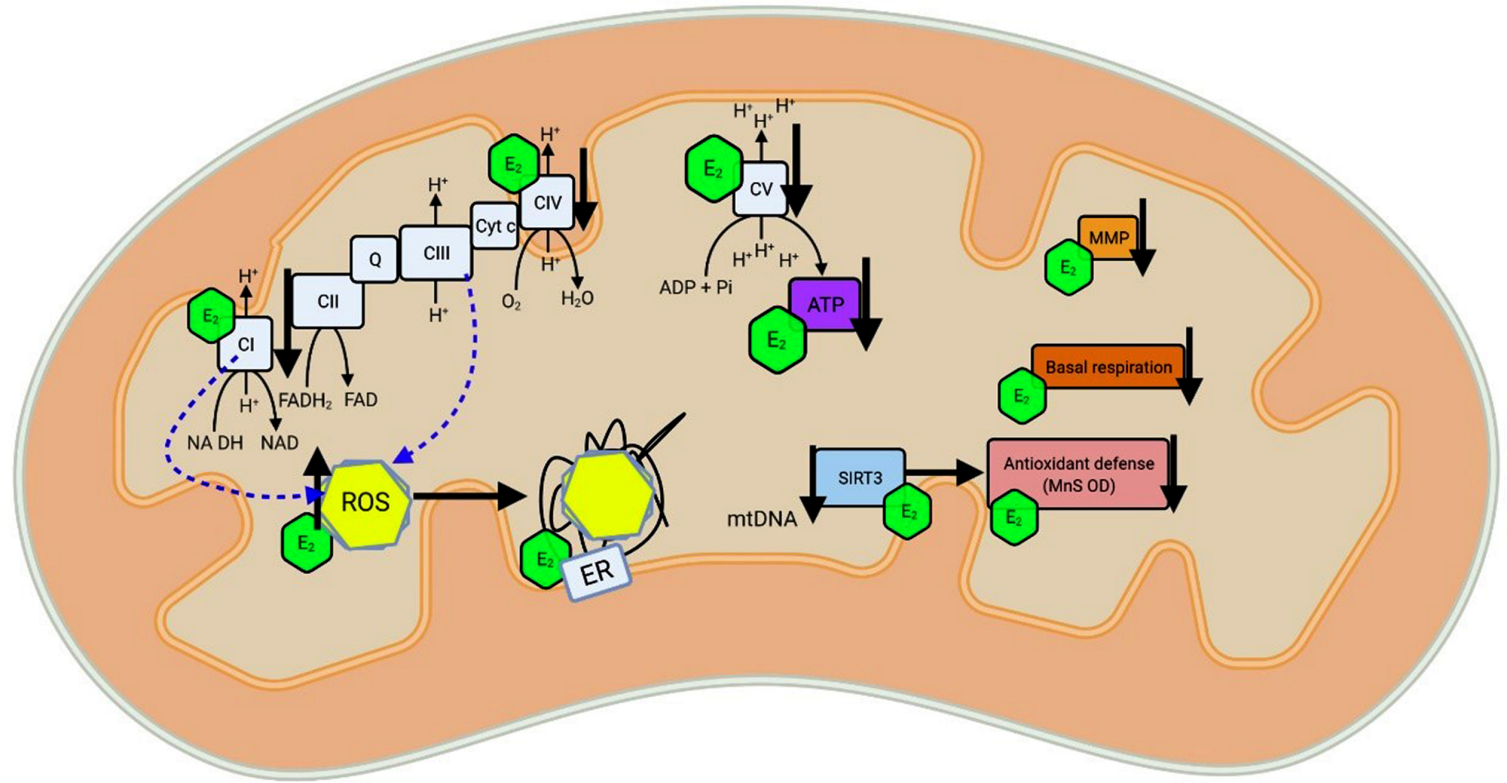
Role of estrogen in affecting mitochondrial function during aging. Aging is associated with a weakening of the electron transport chain (ETC), which decreases ATP levels and mitochondrial membrane potential (MMP). Basal respiration simultaneously decreases antioxidant defenses and increases RNA production by complex I and complexes INAs and MNAs. Estrogen (E2) increases ETC activity, stagnates mitochondrial membrane potential, inhibits reactive oxygen species (ROS) production, and decreases ATP production and basal respiration. Sirtuin 3 (SIRT3) and estrogen can increase mitochondrial activity by enhancing antioxidant defense activity. Estrogen and SIRT3 are recommended to alleviate age-related weaknesses, age-related declines, and age-related events. (*A higher resolution/colour version of this figure is available in the electronic copy of the article*).

Aging is characterized by a severe decline in physiological function, and mitochondrial dysfunction plays an important role in this process, which is also exacerbated by NDDs (Fig. **2**) [110]. Brain energy production is reduced, and redox status changes from an antioxidant to a pro-oxidant state during aging, affecting the partial mitochondrial generation of O_2_ and H_2_O_2_ [111]_._ Healthy young women have higher GSH levels in the frontal and parietal cortex than young men [112]. One of the relevant sources of ROS in the brain is monoamine oxide A (MAO-A), which is present in higher concentrations in the brains of young premenopausal women [113]. An increase in oxidative stress and a decrease in serum estrogen levels were observed in women who underwent bilateral oophorectomy during premenopause. Treatment with estrogen prevented this effect [114]. During normal aging, changes in brain oxidative modifications and mtDNA have been reported [115].

### 4.2 Estrogen in the Central Nervous System

#### 4.2.1 Estrogen, Steroid Synthesis, and Mitochondria

Steroid hormones from peripheral steroid glands are synthesized individually in the nervous system and are called neurosteroids [116]. In the first stage of steroid hormone biosynthesis, which plays a crucial role in the production of mitochondria in pregnancy, the common precursor is steroids and neurosteroids [117]. During steroidogenesis, the limiting step is the transport of cholesterol from outside to inside of mitochondria [118]. In steroid homeostasis, the predominant role of the mitochondrial process is emphasized. The transport of cholesterol is regulated by the interaction between the steroidogenic acute regulatory (StAR) protein and a multicomponent molecular complex (TSPO), the 18-kDa translocator protein [119].

#### 4.2.2 Estrogen: from Brain Development to Brain Aging

Estrogen plays a flourishing role as a pleiotropic factor in the improvement and function of the CNS. There is ample evidence for the development of neural circuits that affects sex hormone production, sex-specific behaviors in both males and females, and the age difference between mammalian brains in adolescence and mammalian females in adulthood [120]. It begins with the formation of the nervous system and continues through adolescence, reproduction, and the early development of brain plasticity in adulthood [121]. Furthermore, memory, cognition, and attention are impaired in many functions of CNS that are associated with hippocampal activity and significantly alter neurosteroids, including estrogen, in peri- and postmenopause due to disturbances in gonadal hormone production [121].

## 5 NEUROLOGICAL DISORDERS

Nearly 50 million people worldwide suffer from NDDs [122]. However, there are no treatments to slow or reverse the neuropathological mechanisms for these disorders [123,124]. Aging is the major risk factor for many NDDs, such as AD, PD, HD, and strokes, leading to gradual and deleterious modifications in the brain. Therefore, to clarify the genetic and cellular basis of NDDs, the aging mechanism must be taken into account [124-126]. Many believe that aging-related changes in cortical bioenergetics and metabolism are fundamental to cognitive impairment [127]. Mitochondria are the main source of energy in the brain that is affected by NDDs in aging [128, 129]. However, there has been no progress in preventing mitochondrial dysfunction that supports memory and brain well-being [18, 130]. Therefore, it is also important to explore the dangerous changes that occur in mitochondria in aging and due to disease or accidents and to improve neurological interventions based on this knowledge [127, 131].

### 5.1 Alzheimer’s Disease

AD is a late-onset NDD characterized by memory loss and deterioration of various cognitive functions [22]. It is expected that by 2050, the number of people aged 85 years or older will increase by approximately 50% (about 370 million) worldwide [132]. AD has become an important health issue, given the large number of older people who will be affected. This debilitating disease urgently requires early diagnosis and preventive and clinical treatments. Extracellular protein deposits (Aß plaques) and neuropathic pathways are pathological features of AD, especially in brain areas associated with learning, memory, and cognition [22, 133, 134]. According to recent biochemical and cellular analyzes in postmortem AD patients and AD transgenic mice, the earliest cellular events for AD development and growth include mitochondrial oxidative disruption and synaptic malfunction.

**Fig. (3):**
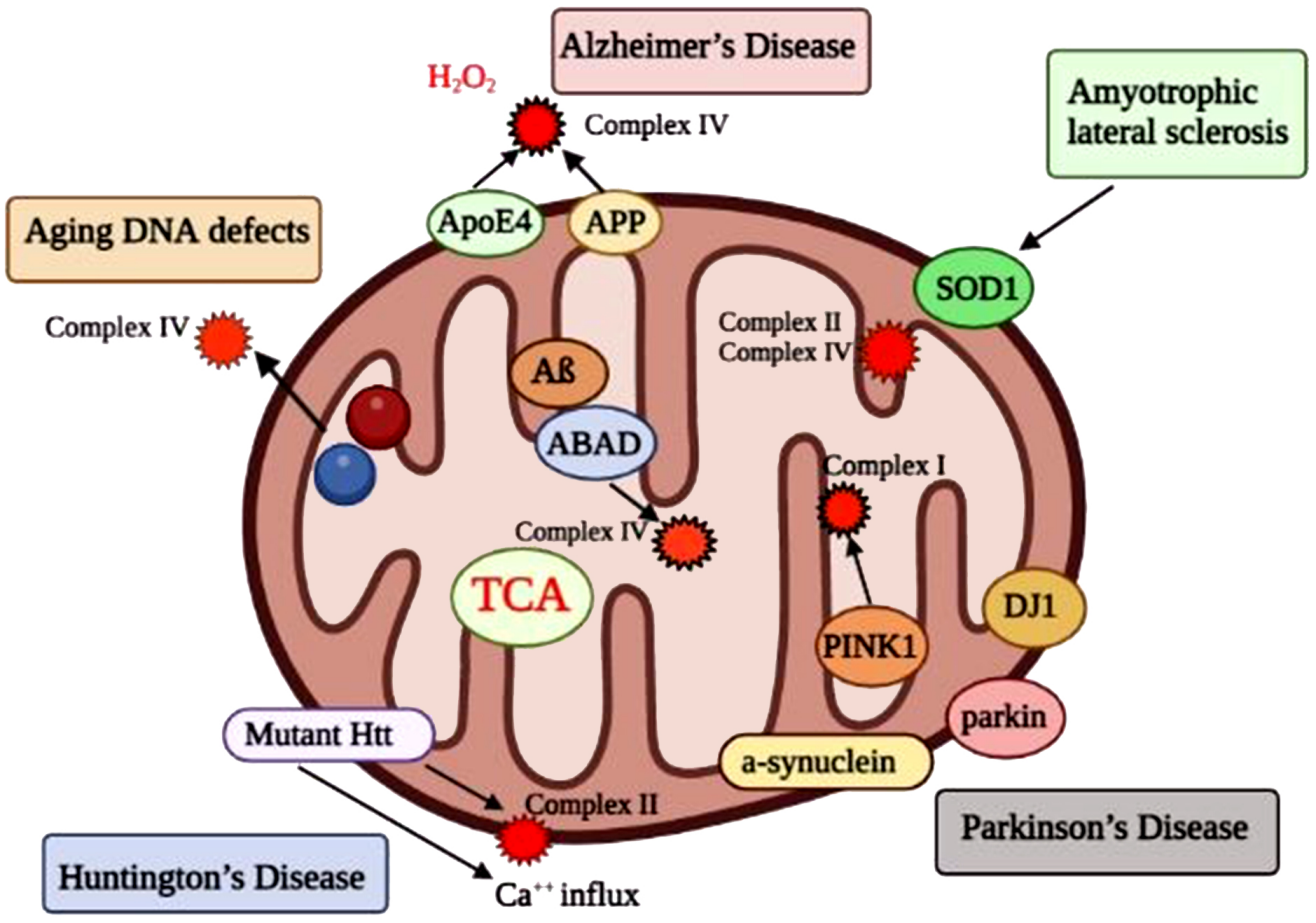
Communication between proteins involved in neurodegenerative defects and mitochondrial disorders. Synthesis of the Aß peptide may contribute to AD due to age-related ROS production and reduced ATP levels. Aß peptides invade mitochondria, induce free radicals, reduce cytochrome oxidase activity, and inhibit the production of ATP. APP is transferred to external mitochondrial membranes in the brains of AD. APP prevents the import of nuclear cytochrome oxidase proteins into mitochondria and may lead to reduced cytochrome oxidase function. Aß is present in the mitochondrial matrix, binds to ABAD, induces free radicals, and causes mitochondrial dysfunction in AD neurons from AD patients, AD transgenic mice, APP cells. The N-terminal segment of ApoE4is associated with mitochondria produces free radicals and causes oxidative damage. Comprehensive proteins, including presenilins, APH, and nicastrin, are included in gamma-secretase in mitochondria, which may lead to the synthesis of Aß and the production of free radicals. Mutant Htt binds HD neurons to the outer membrane of mitochondria and induces free radical formation. Calcium supplementation can also disrupt H_2_O_2_. PD neurons with a-synuclein, PINK1, parkin and DJ1 mutants are associated with mitochondria and induce mitochondrial dysfunction. In PD neurons, the activity of complex I is impaired. In ALS, the SOD1 mutant is located in the internal and external mitochondrial membranes and matrix, causing free and oxidative damage. The deterioration of complex II and IV is associated with ALS. (*A higher resolution/colour version of this figure is available in the electronic copy of the article*).

The molecular mechanisms that cause AD, especially those that require early genetic modification of the disease, are not well understood. Oxidative damage in AD pathogenesis has been identified as an early event [22]. The AD brain is known to have pre-Aß aggregates, tau pathologies, synaptic dysfunction, and inflammatory brain injury [135-138]. Mitochondria from the brain, platelet, and fibroblast of patients with AD have been identified as having oxidative damage. Decreased levels of mitochondrial enzymes were found, including the a-ketoglutarate complex dehydrogenase complex and cytochrome oxidase [139-141]. Markers of oxidative damage were found in the brains of both AD patients and AD transgenic mice. These findings indicate that oxidative damage is vital for the early growth and progression of AD [22]. Recent research in cellular, biochemical and animal models have shown that AD mutants affecting the presenilins, Aß, APP, and ApoE4 are associated with mitochondria and cause mitochondrial dysfunction and oxidative damage in AD (Fig. **3**).

### 5.2 Parkinson’s Disease (PD)

PD is an NDD characterized by muscle spasms, stagnancy, trembling, slow physical movement, and in extreme cases, loss of physical mobility. The pathological changes in PD include the presence of inclusions, or Lewy bodies, and a-synovial in the dopaminergic neurons and cytoplasm of substantia nigra [142-145]. In the last decade, genetic studies have discovered DNA mutations in PD, such as mutations of a-synuclein in the autosomal dominant form of PD [146]. The seminal discovery of mitochondria associated with PD was description of four young patients who rapidly developed symptoms of the disease rapidly after consuming illicit intravenous drug preparation, 1-methyl-4-phenyl-1,2,5,6-tetrahydropyridine. The barrier of complex I found in PD patients induces the formation of free radicals causing oxidants that decrease stress and adenosine triphosphate 9ATP0 [144, 147]. Increased levels of lipid peroxidation markers (4-hydroxnonal) and malondialhydride), protein nitration, and Lewy bodies were found in the substantia nigra [148]. The decrease in glutathione and oxidation levels, which act as an antioxidant, is the earliest identified damage to nigral cells in the brain of PD patients [149].

### 5.3 Huntington’s Disease (HD)

Mitochondrial dysfunction plays a significant role in the pathology of HD [150, 151]. For example, mitochondrial biogenesis has been shown to reduce peroxisome transcription and protein levels in Proliferator-Activated Receptor γ Activator 1α (PGC-17), a key activator protein of mitochondrial biogenesis, based on a postmortem analysis of HD patients with shortness of breath [152, 153]. HD is usually due to genetic mutations resulting in the repetitive CAG coding of polyglutamine within exon 1 of the HD gene. The number of CAG repeats in HD ranges from 3-121 in the affected person, compared to 6-35 in healthy individuals [154]. Huntingtin protein is indispensable for mitochondrial bioenergetics [148].

Interestingly, HD has not only been associated with decreased mitochondrial electronic transport activity [155] but can be modeled in cells and animals by inhibition with 3-nitropropionic acid in complex II [156-158]. Many questions remain, such as the exact process of altered hunting that promotes neurodegeneration and why this disease and the striatum are susceptible to complex II prohibition. This last effect may be due to certain changes in the metabolism of streptomycin, which oxidizes large amounts of lipids and produces more oxidants [159]. Overall, the efficacy of mitochondrial respiratory inhibition in Huntington's strongly suggests that it is of interest for therapeutic advances [160].

### 5.4 Amyotrophic Lateral Sclerosis (ALS)

ALS is a fatal NDD that destroys the neurons controlling voluntary muscles and progresses over time. The upper and lower motor neurons deteriorate or die in ALS, and the instructions they transmit to the muscles are silenced. Muscles that cannot work gradually weaken, atrophy, and twitch. The brain's ability to control voluntary movements eventually deteriorates [161, 162]. ALS patients lose their strength and ability to move their arms, legs, and entire body. ALS affects 1-2 people out of every 100,000 people worldwide, with a uniform distribution and equal proportion across racial groups [163]. The incidence of ALS is highest in people aged 50 to 70 years [162, 164]. Immunoblotting of filter-trappable material revealed accumulations of detergent-insoluble forms of SOD1 in ALS patients [165, 166].

Mitochondrial damage is a common feature of many NDDs and contributes to the degenerative phenotype [167, 168]. Mitochondrial abnormalities ranging from decreased mitochondrial respiration to increased levels of uncoupling proteins have been found in spinal cord and muscle biopsies from ALS patients [169, 170]. Mitochondrial abnormalities have been found in the motor neurons of mice with ALS caused by the accumulation of active dismutase mutants and in animals with hSOD1 G93A mutation, with no loss of motor neurons or other visible damage observed [72, 171]. In certain situations, such as in brain samples from ALS animal models and samples from ALS patients, a fraction of the primary cytosolic SOD1 localizes to mitochondria. In these samples, mutant SOD1 was found in fractions enriched in mitochondria from damaged tissue but not from non-damaged tissue [165, 172-174].

## 6 MITOCHONDRIAL MEDICINE FOR NEUROLOGICAL DISEASES

Similar to nerve cells, mitochondria are essential organelles in eukaryotic cells because they control energy supply, cellular Ca^2+^ homeostasis, apoptosis, and free radicals [12]. The idea of mitochondrial medicine, which refers to medical procedures that target mitochondria, introduces a new direction in biomedical efforts [175]. Disruption of natural mitochondrial activity is detrimental to cell sustainability [176]. Aging and NDDs can be revealed by mitochondrial dysfunction. Mitochondrial dysfunction occurs when germline DNA changes in mtDNA [22]. Neurons are at higher risk for damage in mitochondria. During aging and in other pathological situations, the risk for toxin development increases with the long lifespan of neurons. Disorder of mitochondrial homeostasis can lead to an imbalance of mitochondrial motility, mitochondrial crista lysis, disruption of mitochondrial biosynthesis, pathological mitochondrial autophagy depletion, and mitochondrial oxidative stress [177]. As fascinating as mitochondria are, like any other part of our body, they are susceptible to damage. Heritable mutations in mitochondrial DNA can lead to a variety of different symptoms. This can lead to unusual and extremely rare syndromes, but these are now considered more normal than previously thought [6]. Many NDDs, such as AD, PD, HD, and ALS, involve mitochondrial pathology [12].

### 6.1 Mitochondria-based Interventional Medicine

The genetic material of mitochondria -mitochondrial DNA -encodes the respiratory chain, where oxygen is absorbed and which contains important molecular elements for electron transport [178]. The explanation for the therapeutic utility of mitochondria is that mitochondria play an essential role in the regulation of energy metabolism, development of ROS, and apoptosis [179]. As our understanding of mitochondrial disorders increases, mitochondrial medicine is also rapidly evolving. Mitochondrial interventional medicine, although still in its infancy, is benefiting significantly from the growing awareness of the molecular basis of mitochondrial disorders. The unique manipulation of mitochondria to treat these disorders has demonstrated priorities similar to many existing therapeutic methods that focus only on eradicating mitochondrial dysfunction. The new strategy of mitochondrial medicine can be divided into two groups: (1) prevention of the persistence of mitochondrial disorders; this is of vital importance for the treatment of hereditary mitochondrial disorders such as Leber hereditary optic neuropathy (LHON) (2) manipulation of widespread mitochondrial disorders such as sporadic NDD [180].

Mitochondria-based interventional medicine has emerged and intends to generalize mitochondrial pathology to include cellular bioenergy perturbations, mtDNA mutations, oxidative stress, calcium treatment for impaired mitochondria, mitochondrial apoptosis, and aberrant mitochondrial behavior. However, most of these strategies have not yet been investigated. Nevertheless, with more knowledge about the mystery underlying mitochondrial disorders, the future pattern in mitochondrial treatment will be an interventional medication that directly targets mitochondrial changes [12].

### 6.2 Mitochondria-targeting Therapeutics for Neurodegenerative Diseases

Our insufficient understanding of the etiology of NDDs is a major obstacle to the development of new therapies. Much research indicates that mitochondria play a critical role in age-related NDDs [181]. As mentioned above, early pathogenic events associated with aging and NDDs have demonstrated mitochondrial dysfunction and synaptic damage. These pathogenic events can be treated with: 1) The production of mitochondrial molecules (by targeting ROS) and 2) Therapeutics that increase ATP levels in mitochondrial, which ultimately increases neuronal and synaptic growth [182]. As described above, mitochondrial dysfunctions associated with oxidative stress, generation of a low amount of ATP, mutations of mtDNA, calcium disruptions, and dynamic mitochondrial malfunction are common in NDDs such as AD, PD, HD, and ALS. Removal of oxidants, mitochondrial gene therapy and inhibition of mPTP are the predominant methods for the mitochondrial medication [12].

#### 6.2.1 Redox Therapy

Considering that oxidative stress is one of the most common mitochondrial disorders leading to neurodegenerative defects and the benefits of an antioxidant strategy, research on scavengers of oxygen-free radicals, in addition to oxidative phosphorylation, synthesis of ATP, homeostasis of calcium, production of ROS, and apoptosis, has provided a therapeutic solution for NDDs [12]. Nevertheless, oxidative stress affects the redox balance of cells as a whole [183]. Certain epidemiological studies suggest that increased consumption of antioxidant vitamins, including vitamin E, vitamin C, and beta-carotene, may minimize the risk of AD or PD. Available antioxidants are currently ineffective in treating neurodegenerative conditions because the BBB is not crossed by naturally occurring antioxidants such as vitamins E and C, so free radicals do not have access. Improved delivery of antioxidants to the brains of NDD patients is important to address these complications and to determine whether antioxidants might be useful as therapeutics [48]. In general, these findings indicate a promising treatment pathway for the therapy of NDDs through the delivery of antioxidants that protect mitochondria and minimize the adverse events associated with oxidative stress [181].

#### 6.2.2 Mitochondrial Permeability Transition Pores Inhibition

Formation of mitochondrial permeability transition pores (mPTP) decreases mitochondrial membrane potential, increases ROS formation, triggers calcium perturbations in mitochondria, and promotes the proapoptotic release of mitochondrial molecules. Extreme mitochondrial dysfunction mediated by mPTP is thought to exacerbate pathological neuronal injury with previously identified neurodegeneration [12]. Interest in mPTP increased even further when the immunosuppressive agent cyclosporine A (CSA) was shown to directly block the mPTP [184]. This mPTP inhibition by CsA is accompanied by its association with cyclophilin D (CypD) of the mitochondrial protein matrix, preventing its association with the ANT [185]. The ANT is a vital IMM protein of the solute transporter family that mediates the exchange of ADP for ATP between the cytosol and the mitochondrial matrix [186]. Animal models of various NDDs have investigated the protective effects of mPTP blockade, and recent studies have focused on the inhibition of CypD or voltage-dependent anion channel (VDAC) (Table **1**) [12, 187-191].

**Fig. (4):**
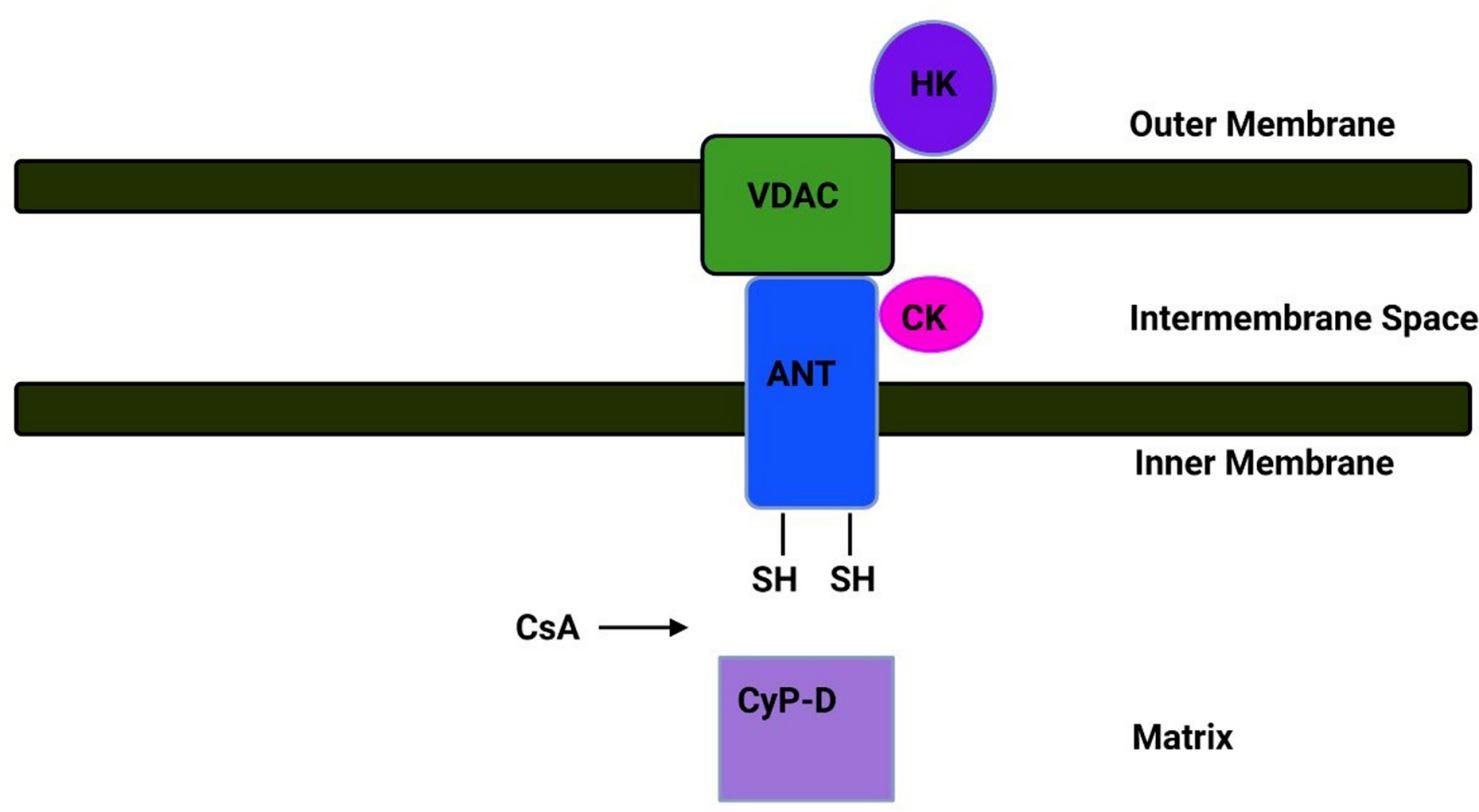
Proposed PT pore structure. CsA blocks the opening of the pores by inhibiting the binding of cyclophilin-D (CyP-D) to the adenine nucleotide translocator (ANT). PTPC is characterized as a voltage-dependent polyprotein complex that, under certain conditions, can form a nonselective channel at the contact sites between the two mitochondrial layers. Mitochondria contain all the proteins essential for mPTP acceptance, so mPTP does not require biosynthesis. The physiological role of mPTP opening needs further investigation. Be that as it may, the emerging information suggests that concentrating on mPTP opening ought to be done with caution. **Abbreviations**: VDAC (voltage-dependent anion channel); HK, (hexokinase); CK, (creatine kinase). (*A higher resolution/colour version of this figure is available in the electronic copy of the article*).

VDAC is a protein of 32 kDa that exists as a dimer at the outer membrane of mitochondria and allows the entry and exit of various metabolites important for the metabolism of mitochondria [192]. While VDAC has been shown to interact with ANT during pore development, its involvement in the pore has recently been questioned because mitochondria isolated from VDAC knockout mice are exposed to the same degree of Ca^2+^-induced swelling of mitochondria (Fig. **4**) [193].

#### 6.2.3 Mitochondrial Gene Therapy

For physicians in all medical fields, the information gathered over the past twenty years in patients with mitochondrial diseases is of great importance [194]. The majority of mitochondrial functional proteins are encoded by the nuclear genome (nDNA), and more than 1,500 nDNA genes are known to be involved in mitochondrial function and structure [195]. In NDDs, the frequent occurrence of mtDNA defects calls for research to reduce the lesion of mtDNA, especially in genetic modification of mtDNA and downstream genes. Some successful discoveries have been made, which are classified into three classes: selective inhibition of mtDNA mutants, recombinant replacement of mtDNA, and allotropic expression of the mitochondrial protein [12]. mtDNA also coexists with wild-type genomes with pathogenic mutations (mtDNA heteroplasmy). Mitochondrial dysfunction and disease occur only when there are high numbers of mutant mtDNAs, and thus reduction of mtDNA mutants may be an appropriate treatment for this condition [196]. The two well-studied tactics for selective mtDNA inhibition are the removal of mtDNA by limiting endonuclease collection and the blockade of mutant mtDNA by antisense RNA. COX PstI, COX8-ApaLI, and ScaI have been found to restore mitochondrial function [12]. Recently, it has been reported that sporadic PD is associated with mitochondrial dysfunction, especially a proton shift in NADH-quinone oxidoreductase (complex I). Indeed, many PD patients reported that they have less difficulty following the treatment. Moreover, Parkinson's symptoms have been shown to be caused by complex I inhibitors such as MPTP [197]. Substitution of the defective mtDNA with a “healthy” recombinant mtDNA genome is beneficial not only for heteroplasmic but also for homoplasmic patients. Although there have been some tests of the therapeutic efficacy of recombinant mtDNA, the low accessibility of human mtDNA constructs, the weak mitochondrial import of large structures, and the competition of recombinant mtDNA with mtDNA as a functional resident significantly limit the use of this approach as a treatment strategy [12]. The first active gene therapy based on *in vivo* treatment with mtDNA gave hope for the treatment of mitochondrial disorders. Gene therapy has shown promising effects in the treatment of NDDs. In addition, further theoretical cures for mitochondrial disorders are planned [198].

### 6.3 Targeting ROS Production

ROS is a colloquial term for an active compound group that contains oxygen generated from aerobic metabolism [199]. Oxidative stress can occur when ROS oxidation outweighs antioxidation, which is thought to result in cellular damage [200]. The brain has the most active oxidative metabolism with a high oxygen demand compared to other organs [201]. ROS is associated with cell damage and traditionally acts only as unfriendly molecules, although ROS exposure is inevitable for aerobic cells [202, 203]. The major source of intracellular ROS is now well documented; 90% originates from the air chain of the inner mitochondrial membrane. With the formation of O^2-^ by the combination of leak electrons from the respiratory complexes of mitochondria (mainly complexes I and III), mitochondrial ROS formation is initiated. In the presence of SOD, highly active O^2-^ can then be converted to stable H_2_O_2_ [204]. ROS emissions into mitochondria are very low under normal circumstances and cause minimal damage because mitochondria have robust antioxidant protection systems that adequately scavenge unneeded ROS. The “ROS-induced ROS release” (RIRR) is a positive feedback mechanism responsible for the ROS-mitochondria interaction. An explosion of mitochondrial ROS is triggered during RIRR, which decreases MMP and allows a longer opening of the mPTP [205]. As mentioned earlier, abnormally high ROS levels can be rapidly neutralized to cellular concentrations by a complex network of various robust antioxidants, which are necessary for the maintenance of normal cellular functions [206].

Neuronal deficiency is a central component in the pathophysiological mechanism of oxidative stress, mitochondrial disorders, pathological protein accumulation, and so on in nervous system diseases [207]. Oxidative stress or ROS is one of the most important problems [204]. As we know, the brain weighs only 2% of the body weight. Nevertheless, metabolic oxygen uptake accounts for 20% of the body's total oxygen consumption under non-stress conditions, and more ROS often follows strong demand for oxygen [204]. The Nrf2/ARE pathway is recognized as an effective defense system in oxidative stress, which would provide a viable avenue for antioxidant treatment in ischemia-reperfusion (I/R) injury [208]. However, the disease may also be exacerbated by a substantial decrease in glutathione peroxide (GPx) and chloramphenicol acetyltransferase (CAT) levels, as well as total GSH levels, indicating a weak early AD antioxidant protective mechanism [209].

### 6.4 Triphenylphosphonium Cation-Based Antioxidants

Triphenylphosphonium (TPP) has been widely used as a lipophilic cation in the development of mitochondrial antioxidants. In conjunction with TPP mobility, antioxidants can achieve more than a 1000-fold increase in mitochondrial concentration, depending on the capacity of the cell membrane and mitochondrial membranes [210]. The cation of lipophilic triphenylphosphonium is bound to antioxidants, including vitamin E and coenzyme Q, and these antioxidants bound to the triphenylphosphonium cation preferentially occupy mitochondria due to the charge difference between mitochondria (with a high negative charge) and antioxidants dependent on the lipophilic triphenylphosphonium cation (with a high positive charge) [22].

#### 6.4.1 MitoQ

MitoQ is a powerful antioxidant for mitochondrial maintenance that has been successfully used. MitoQ contains two redox derivative formulations of ubiquinone that target mitochondria: mitoquinol reduction and mitoquinone oxidization [211]. MitoQ is part of the lipid-buried center of the respiratory chain of the inner mitochondrial membrane, where two electrons are accepted from complexes I and II to form the reduction compound ubiquinol, which donates electrons to complex III [212]. MitoQ can excessively accumulate H_2_O_2_ in mitochondria and convert it to H_2_O and O_2_ and minimize the toxicity caused by mitochondrial free radicals [22].

#### 6.4.2 MitoVitE

MitoVitE is an antioxidant that contains triphenyl-phosphonium bromide (TPPB) and [2-3,4-dihydro-6-hydroxy-2,5,7,8tetra-methyl-2H-1-benzopyran-2-yl] [29]. MitoVitE, a derivative of vitamin E, was produced to study the toxicity of mitochondria and cells. MitoVitE, like MitoQ, is readily taken up into mitochondria [22]. MitoVitE was found to decrease caspase activity induced by H_2_O_2_ and prevent stress-induced oxidative death of a cell in FDRA patients with cultured fibroblasts [213]. MitoVitE decreases ethanol-induced intracellular oxidant aggregation and preserves glutathione peroxidase/glutathione reductase functions, gamma-glutamylcys-teine synthetase protein expression, and total cellular glutathione levels [214]. In the elderly and people with NDDs, further studies are needed to investigate the use of MitoVitE [22].

#### 6.4.3 MitoPBN

MitoPBN is an antioxidant containing triphenylphosphonium bromide and [4-[4 (1,1-dimethyl ethyl) oxidoimino]––methyl]phenoxy]butyl]. PBN has been shown to protect animal ischemia models from endotoxin shock and from ailments that can induce mitochondrial toxicity and neurodegeneration [215]. PBN shows high reactivity with carbon-centered radicals [216]. MitoPBN is readily taken up by mitochondria, similar to MitoQ and MitoVitE [22].

## CONCLUSION AND FUTURE PERSPECTIVE

Mitochondria are important organelles essential for neuronal longevity through ATP production. Impairment of mitochondria in terms of increased production of ROS, alteration of mitochondrial conductance, deterioration of membrane potential in mitochondria, and decreased synthesis of mitochondrial ATP contributes to extreme neural degradation and consequently causes neuronal death. Many areas of research show that mitochondria contribute to age-related NDDs, such as AD, PD, HD, and ALS. Several methods for treating mitochondrial disorders that are increasingly used are reviewed in this paper, namely 1) Mitochondria-based interventional medicine, 2) Mitochondria-targeting therapeutics for NDDs (redox therapy, inhibition of mitochondrial permeability transition pores, mitochondrial gene therapy), 3) Targeting ROS production, and 4) Triphenylphosphonium cation-based antioxidants (MitoQ, MitoVitE, MitoPBN). Studies suggest that mitochondrial dysfunction is strongly implicated in the pathophysiological mechanisms of neurodegeneration. Therefore, mitochondria-targeting drugs are highly promising as a potential prevention strategy in NDDs. Antioxidant therapy has brought mitochondria-targeting drugs into the spotlight. Several drugs are currently being tested in clinical testing in people with NDDs or other diseases and have been shown to be highly effective in medical use. Finally, mitochondrial therapy targeting the underlying pathology of early-stage neurodegeneration is a potential therapeutic approach to treat NDDs. NDDs are dynamic diseases that require interdisciplinary investigation. Rapid implementation of the system and technology would require quantitative analysis of mitochondrial malfunction *in vitro* and *in vivo*. It would identify the impact of mitochondrial dysfunction on the development of the disorder in neurodegenerative models.

## Figures and Tables

**Table 1:** Pharmacological report of the mPTP inhibitors.

**mPTP Inhibitors**	**Interaction**	**Effect**	**References**
Cyclosporin A (CsA)	CypD	Inhibition of MPTP, decreasing sensitivity to calcium ions, in the central nervous system (CNS)	[188, 189]
ANT inhibitor	Bongkrekic acid	Decreased ROS production in mitochondria	[190]
VDAC1	Frontal cortex	VDAC1 level is decreased and causes Alzheimer´s disease	[191]
VDAC2	Temporal cortex	VDAC2 level is increased and causes Alzheimer´s disease	[191]

**Note**: [187]{Reddy, 2009 #115}.

## LIST OF ABBREVIATIONS

mtDNA: Mitochondrial DNA
NDDs: Neurodegenerative Disorders
AD: Alzheimer’s Disease
PD: Parkinson’s Disease
HD: Huntington’s Disease
ALS: Amyotrophic Lateral Sclerosis
ATP: Adenosine Triphosphate
mRNAs: Messenger Ribonucleic Acids
OMM: Outer Mitochondrial Membrane
IMS: Intermembrane Space
VDACs: Voltage-dependent Anion Channels
RONS: Reactive Oxygen and Nitrogen Species
ROS: Reactive Oxygen Species
VDAC: Voltage-dependent Anion Channel
